# Effects of olanzapine combined with sodium valproate on symptoms, inflammatory markers, and cognitive function in patients with bipolar disorder

**DOI:** 10.1097/MD.0000000000042803

**Published:** 2025-06-27

**Authors:** Xiao Wang, Fuyu Ye, Zhen Zhang, Qigui Zhang, Guanhua Li

**Affiliations:** a Department of Psychological Assessment Room, Jiujiang Fifth People’s Hospital, Jiujiang, Jiangxi Province, China; b Functional Inspection Room, Jiujiang No. 1 People’s Hospital, Jiujiang, Jiangxi Province, China; c Department of Psychiatry, The Fifth People’s Hospital of Jiujiang City, Jiujiang, Jiangxi Province, China; d Department of Clinical Laboratory, Jiujiang City Key Laboratory of Cell Therapy, Jiujiang No. 1 People’s Hospital, Jiujiang, Jiangxi Province, China.

**Keywords:** bipolar disorder, depression, inflammatory markers, mania, olanzapine, sodium valproate

## Abstract

Bipolar disorder (BD) is a chronic mental health condition characterized by alternating manic and depressive episodes that significantly impact mood and cognitive function. Recent research has linked BD to neuroinflammation. Despite the availability of antipsychotics and mood stabilizers as standard treatments, their efficacy and side effects can vary, underscoring the need for more effective therapeutic strategies. This study aims to evaluate the combined effects of olanzapine and sodium valproate on clinical symptoms and inflammatory markers in patients with BD. A total of 80 BD patients from the Fifth People’s Hospital of Jiujiang, treated between April 2022 and April 2024, were enrolled in the study. They were divided into 2 groups: the control group, which received olanzapine monotherapy, and the observation group, which received a combination of olanzapine and sodium valproate. After 8 weeks of treatment, clinical efficacy was assessed using the Bech-Rafaelsen Mania scale and the Hamilton depression scale. Serum inflammatory markers, including interleukin-1, interleukin-10, and tumor necrosis factor-alpha, were measured along with neuroendocrine function indicators (thyroid hormone, cortisol, thyroid-stimulating hormone, and adrenocorticotropic hormone). Cognitive function was evaluated using computerized assessments, and adverse events were monitored and recorded. The observation group exhibited significantly lower Bech-Rafaelsen Mania scale and Hamilton depression scale scores, as well as reduced interleukin-1 and tumor necrosis factor-alpha levels compared to the control group. Both groups showed decreased levels of serum thyroid hormone, cortisol, and adrenocorticotropic hormone, along with increased thyroid-stimulating hormone levels after treatment, with more pronounced changes observed in the observation group (*P* < .01). Additionally, cognitive function scores were significantly higher in the observation group (*P* < .05), and the incidence of adverse events was lower (15.00% vs 22.50%, χ² = 0.38, *P* < .01). The combination of olanzapine and sodium valproate leads to significant improvements in clinical symptoms, reduces inflammatory markers, enhances cognitive function, and demonstrates greater safety compared to olanzapine monotherapy. This combination therapy offers a promising treatment approach for managing BD.

## 1. Introduction

Bipolar disorder (BD) is a chronic psychiatric condition characterized by recurrent mood episodes, including manic and depressive phases. These mood fluctuations can significantly impair daily functioning and quality of life. BD affects approximately 1% to 4% of the global population and is associated with a high risk of suicide, with individuals experiencing manic or depressive episodes being 20 to 30 times more likely to die by suicide compared to the general population.^[1–3]^ The substantial impact of BD on individuals and society underscores the need for effective treatment strategies that not only target the symptoms but also mitigate potential long-term complications, such as cognitive decline and neuroinflammation, which are increasingly recognized as key contributors to the disorder’s pathophysiology.^[4]^

Pharmacological management of BD relies heavily on mood stabilizers and antipsychotics, with olanzapine being a first-line option due to its efficacy in stabilizing mood and preventing relapse.^[5]^ However, its utility is limited by side effects, particularly in early-onset BD.^[6]^ While alternative treatments exist, olanzapine remains widely prescribed for refractory cases, necessitating strategies to mitigate its adverse effects.^[7]^

The combination of olanzapine with sodium valproate has emerged as a promising therapeutic strategy for BD. Sodium valproate, traditionally used as an anticonvulsant, also serves as an effective mood stabilizer, particularly in patients who experience frequent mood swings. Valproate role in modulating neuroinflammatory processes and its potential for protecting against cognitive dysfunction have made it a valuable adjunctive treatment in BD.^[8,9]^ Recent studies suggest that the combination of olanzapine and sodium valproate might reduce some of the adverse effects associated with olanzapine alone, while enhancing the treatment’s overall efficacy.^[10,11]^

Neuroinflammation has been implicated in the pathophysiology of BD, with increased levels of pro-inflammatory cytokines such as interleukin-1 (IL-1) and tumor necrosis factor-alpha being observed in patients with BD, particularly during manic episodes.^[12,13]^ These inflammatory markers are thought to play a critical role in the neurobiological mechanisms that contribute to mood episodes, cognitive dysfunction, and other symptoms of BD. Recent research has also highlighted the impact of antipsychotic and mood stabilizer treatments on inflammatory markers, suggesting that reducing inflammation could be crucial for improving clinical outcomes and preventing long-term cognitive decline in BD patients.^[14,15]^

In addition to inflammatory changes, cognitive dysfunction is a significant concern in BD, with deficits in attention, memory, executive function, and processing speed commonly observed. Cognitive impairment in BD is associated with poor functional outcomes, including difficulty in maintaining employment and relationships.^[16]^ While cognitive deficits are often considered secondary to mood symptoms, recent studies emphasize the need to address cognitive dysfunction directly in the treatment of BD, as these impairments are often persistent and may remain even during periods of mood stabilization.^[17,18]^

Given the potential of both olanzapine and sodium valproate in addressing both the core symptoms of BD and the accompanying neuroinflammation and cognitive dysfunction, this study aims to evaluate the combined effects of these 2 medications on clinical symptoms, inflammatory markers, and cognitive function in BD patients. We hypothesize that the combination therapy will not only improve mood symptoms and reduce inflammatory markers but also enhance cognitive function more effectively than olanzapine monotherapy, providing a safer and more comprehensive treatment approach for BD patients. This study will contribute to the growing body of evidence supporting combination therapies in BD, particularly in improving both short-term and long-term outcomes.

## 2. Materials and methods

### 2.1. General information

This study was approved by the Ethics Committee of the Fifth People’s Hospital of Jiujiang City. This study included 80 patients diagnosed with BD at the Fifth People’s Hospital of Jiujiang between April 2022 and April 2024. The patients were divided into 2 groups based on the treatment they received: a control group and an observation group, with 40 patients in each. The age range of the included patients was limited to 18 to 60 years. Inclusion criteria required patients to have a Bech-Rafaelsen mania scale (BRMS)^[19]^ score above 5 and a Hamilton depression scale (HAMD)^[20]^ score of no <17.

During the manic episode: patients with significant manic symptoms were assessed using the BRMS. During the depressive episode: patients with significant depressive symptoms were assessed using the HAMD. Throughout the entire course of the illness, if a patient experienced both manic and depressive states, the appropriate scale was used during the respective episodes (BRMS during the manic phase and HAMD during the depressive phase). Each hospitalized patient might experience multiple hospitalizations and different emotional states during the course of the illness; therefore, the appropriate scale was selected for assessment based on the clinical manifestations at each hospitalization. It was also ensured that the patients’ major organ functions were normal.

The exclusion criteria were strict, including patients with other mental disorders, tumors, organic diseases, alcohol or drug dependence, severe cardiovascular and cerebrovascular diseases, those who had received treatment within 1 month prior to the study, those who were breastfeeding or pregnant, and those allergic to the study drugs. Statistical analysis showed no significant differences in general data between the 2 groups (*P* > .05). Data are detailed in Table 1.

**Table 1:** Comparison of general data between the 2 groups.

Project	Control group (n = 40)	Observation group (n = 40)	*χ*^2^ (*t*)	*P*
Gender/cases				
Male	22	21	0.16	.685
Female	18	19		
Age ($\bar{x}\pm s$), yr	35.79 ± 4.26	35.62 ± 4.30	0.18	.848
Course of disease ($\bar{x}\pm s$), yr	2.05 ± 0.59	2.03 ± 0.57	0.23	.822
Educational level/cases				
Junior high school and below	14	16	0.15	.901
High school or vocational school	13	12		
Junior college or above	13	12		

This study included 80 patients diagnosed with BD at Jiujiang Fifth People’s Hospital between April 2022 and April 2024. The patients were divided into 2 groups based on their treatment plans: a control group and an observation group, with 40 patients in each. The inclusion criteria required patients to be aged between 18 and 60 years, with a score higher than 5 on the BRMS and not <17 on the Hamilton depression scale (HAMD). Assessments were made during manic episodes using the BRMS and during depressive episodes using the HAMD. Patients with comorbid mental disorders, organic diseases, substance dependence, severe cardiovascular diseases, or those who had received treatment within 1 month prior to the study were excluded. Both groups showed no significant differences in general demographic and clinical characteristics (*P* > .05), ensuring comparability between the groups. Detailed data is provided in Table 1.

### 2.2. Treatment methods

Both groups received standardized psychological treatments, including cognitive behavioral therapy and family therapy, alongside psychological support services, regardless of whether the patients were in the manic or depressive phase. In both phases, the control group received standard treatment combined with Olanzapine (10 mg daily), while the observation group received a combination of Olanzapine (10 mg daily) and Sodium Valproate. The initial dose of Sodium Valproate was set at 1000 mg/day. After 1 week, the dose was adjusted based on the patient’s clinical response and tolerability. Adjustments were made according to 3 main factors: clinical symptoms, such as residual mood or behavioral symptoms; side effects, including gastrointestinal disturbances or tremors; and laboratory values, specifically monitoring the serum sodium valproate levels to maintain a therapeutic range of 50 to 100 μg/mL. If necessary, the dose was gradually increased up to a maximum of 30 mg/kg/day. Both groups completed an 8-week treatment period.

### 2.3. Observation indicators and methods

Symptom assessment: The BRMS and HAMD scales were used to assess manic and depressive symptoms, respectively, before and after the 8-week treatment. BRMS has 13 items scored from 1 to 4, with a maximum score of 52, and HAMD has 17 items with a total possible score of 56. Higher scores indicate more severe symptoms. A reduction of ≥ 50% in the BRMS score compared to the baseline is considered as a treatment response for manic symptoms. Similarly, a reduction of ≥ 50% in the HAMD score compared to the baseline is considered a treatment response to depressive symptoms. Inflammatory markers: fasting venous blood samples were collected from both groups before and after treatment to measure serum levels of IL-1, IL-10, and TNF-α using ELISA. Neuroendocrine function: radioimmunoassay was used to accurately measure key neuroendocrine indicators, including thyroid hormone (TH), cortisol (Cor), thyroid-stimulating hormone (TSH), and adrenocorticotropic hormone (ACTH). Safety evaluation: adverse reactions, such as orthostatic hypotension, dizziness, thrombocytopenia, and dry mouth, were recorded to ensure data rigor and integrity.

#### 2.3.1. Cognitive assessment

To assess cognitive function, a comprehensive neuro-psychological battery was employed before and after the 8-week treatment period. The cognitive assessments included:

Executive function: The Stroop color-word task was used to evaluate the ability to inhibit automatic responses and manage cognitive flexibility in the presence of conflicting information.

Working memory: The digit span backward and forward tasks were used to assess the capacity for maintaining and manipulating information in short-term memory.

Visual memory: The spatial span backward task and the spatial span task were utilized to assess visual-spatial working memory and attention span.

Processing speed: The coding test and symbol search tasks were used to evaluate the speed of processing visual information and performing cognitive tasks under time constraints.

Attention: The rapid judgment task was employed to measure attention and the ability to process information quickly.

These tasks were selected for their relevance and reliability in assessing the key cognitive domains most affected by BD. Computerized versions of the tasks were used to ensure precision and consistency in cognitive assessment.

### 2.4. Statistical methods

Data were rigorously analyzed using SPSS 25.0 software (Chicago). Continuous variables were expressed as mean ± standard deviation ($\bar{x}\pm s$) and analyzed using the t-test, while categorical data were expressed as numbers and percentages (%) and analyzed using the chi-square (χ^2^) test. A *P*-value of < .05 was considered statistically significant.

## 3. Results

### 3.1. Manic and depressive symptom scores

After 8 weeks of treatment, the BRMS scores in the observation group significantly decreased from 15.13 ± 2.26 to 6.39 ± 1.21, with a statistically significant difference compared to the control group (*P* < .001). The control group also showed a reduction in BRMS scores, from 15.63 ± 2.37 to 8.73 ± 1.25. Notably, the BRMS score in the observation group was significantly lower than that of the control group after treatment, with the difference being statistically significant (*P* < .001). Furthermore, 28 (70%) patients in the observation group achieved a therapeutic response (≥50% reduction), compared to 12 (30%) in the control group (*P* < .001), indicating a more favorable outcome in the observation group.

Similarly, the Hamilton depression scale (HAMD) scores decreased significantly in both groups. In the observation group, the HAMD score dropped from 19.13 ± 2.24 to 15.56 ± 1.95, while in the control group, it decreased from 26.33 ± 3.47 to 23.63 ± 3.26. The observation group’s HAMD score was significantly lower than that of the control group after 8 weeks of treatment (*P* < .001). Additionally, 30 (75%) patients in the observation group showed a therapeutic response (≥50% reduction) in HAMD scores, compared to 8 (20%) in the control group (*P* < .001), highlighting a more substantial therapeutic effect in the observation group. These findings are summarized in Table 2.

**Table 2:** Comparison of manic and depressive symptom scores between 2 groups ($\bar{x}\pm s$).

Project	Control group (n = 40)	Observation group (n = 40)	*t*/χ²	*P*
BRMS score				
Before treatment	15.63 ± 2.37	15.13 ± 2.26	0.19	.709
Treatment for 8 wk	8.73 ± 1.25	6.39 ± 1.21	7.19	<.001
Therapeutic response (≥50% reduction)	12 (30%)	28 (70%)	12.45	<.001
HAMD score				
Before treatment	26.33 ± 3.47	19.13 ± 2.24	0.12	.891
Treatment for 8 wk	23.63 ± 3.26	15.56 ± 1.95	6.37	<.001
Therapeutic response (≥50% reduction)	8 (20%)	30 (75%)	20.45	<.001

BRMS = Bech-Rafaelsen mania scale, HAMD = Hamilton depression scale.

### 3.2. Cognitive function

The comparison of cognitive assessment items before and after treatment between the 2 groups showed that by the eighth week after treatment, the observation group had a significantly higher verbal working memory digit forward score (1.40 ± 0.60) and a lower digit backward score (7.25 ± 1.94) compared to before treatment, with statistically significant differences (*P* < .05). In visual memory, the spatial span backward (4.11 ± 0.70) and spatial span forward (6.13 ± 1.03) scores were both lower than before treatment, with the spatial span forward score showing a significant difference (*P* < .05). For processing speed, the symbol search (100.52 ± 13.88) and coding test (94.71 ± 9.17) scores were higher, while the trail making test (45.11 ± 5.80) score was lower than before treatment (*P* < .05). In terms of executive inhibition, the Stroop word task score (304.88 ± 32.65) was higher than before treatment (*P* < .05). When comparing between groups, the observation group had higher scores in digit forward and the coding test at the eighth week after treatment compared to the control group (*P* < .05) (see Table 3).

**Table 3:** Comparison of 2 cognitive assessment items ($\bar{x}\pm s$).

Project	Observation group (n = 40)		Control group (n = 40)	
	Before treatment	Treatment for 8 wk	Before treatment	Treatment for 8 wk
Verbal working memory				
Number front back	0.40 ± 0. 11	1.50 ± 0.60*	0.45 ± 0.10	1.45 ± 1.64
Reverse numbers	27.60 ± 1.88	7.25 ± 1.94**	27.64 ± 1.73	7.70 ± 1.71
Visual memory				
Reverse spatial breadth	20.25 ± 2.72	4.11 ± 0.70	20.21 ± 2.56	4.24 ± 2.56
Room size	30.90 ± 1.04	6.13 ± 1.03**	30.93 ± 1.02	6.96 ± 1.01
Processing speed				
Symbol search	44.60 ± 12.87	100.52 ± 13.88**	45.24 ± 12.68	95.35 ± 12.53
Decoding test	36.03 ± 5.83	94.71 ± 9.17*	34.01 ± 5.14	93.01 ± 15.00
Sequential connection	84.94 ± 9.75	45.11 ± 5.80**	89.34 ± 7.91	46.56 ± 7.63
Execution inhibition				
Stroop word color	43.76 ± 12.00	212.00 ± 24.62	43.75 ± 1.98	203.86 ± 31.93
Stroop words	78.91 ± 11.33	304.88 ± 32.65**	79.00 ± 11.19	228.28 ± 21.03
Attention				
Quick judgment	69.53 ± 8.79	227.62 ± 18.55	69.39 ± 8.61	209.19 ± 21.61

* Compared with the control group, *P* < .05.

** Compared with before treatment, *P* < .05.

### 3.3. Comparison of neuroendocrine function indicators

When comparing the changes in serum indicators before and after treatment, this study found that there was no statistically significant difference in the levels of serum TH, Cor, TSH, and ACTH between the 2 groups in the initial stage (*P* > .05). However, after 8 weeks of treatment, retesting showed that the serum levels of TH, Cor, and ACTH in both groups decreased compared to before treatment, while the serum TSH levels increased. It is worth noting that the changes in these indicators, i.e. the degree of decrease or increase, in the observation group were significantly greater than those in the control group, and this difference was highly significant statistically (*P* < .01) (see Table 4).

**Table 4:** Comparison of neuroendocrine function indicators between 2 groups before and after treatment ($\bar{x}\pm s$).

Group	Time	TH (ng/mL)	Cor (nmol/L)	TSH (mU/L)	ACTH (ng/L)
Control group (n = 40)	Before treatment	88.10 ± 1.51	301.23 ± 9.45	2.01 ± 0.16	77.03 ± 7.05
	Treatment for 8 wk	83.40 ± 1.47	259.31 ± 8.48	3.05 ± 0.26	51.27 ± 5.38
Observation group (n = 40)	Before treatment	88.29 ± 2.17	288.32 ± 8.05	2.03 ± 0.27	75.27 ± 6.03
	Treatment for 8 wk	78.03 ± 1.14	204.26 ± 7.03	3.78 ± 0.48	41.27 ± 4.01
*t*/*P* control group (before and after treatment)		7.303/<.001	14.601/<.001	11.325/<.001	12.422/<.001
*t*/*P* Observation group (before and after treatment)		14.758/<.001	36.461/<.001	12.547/<.001	20.085/<.001
*t*/*P* Inter group (after treatment)		13.488/<.001	19.041/<.001	4.630/<.001	5.515/<.001

ACTH = adrenocorticotropic hormone, Cor = cortisol, TH = thyroid hormone, TSH = thyroid-stimulating hormone.

### 3.4. Inflammatory indicator levels

There was no significant difference in the levels of IL-1, IL-10, and TNF-α in serum inflammatory factors before and after treatment (*P* > .05). After 8 weeks of treatment, the levels of IL-1 and TNF-α significantly decreased, while the level of IL-10 significantly increased. The changes in inflammatory factors in the observation group were more significant (*P* < .05 or *P* < .01) (see Table 5).

**Table 5:** Comparison of inflammatory index levels between 2 groups ($\bar{x}\pm s$) ng/L.

Group	Time	IL-1	IL-10	TNF-α
Control group (n = 40)	Before treatment	103.56 ± 15.12	148.31 ± 24.06	152.20 ± 28.38
	Treatment for 8 wk	88.48 ± 11.02	168.45 ± 27.37	125.07 ± 25.38
Observation group (n = 40)	Before treatment	114.35 ± 14.23	150.16 ± 22.34	152.33 ± 30.04
	Treatment for 8 wk	70.22 ± 7.45	199.55 ± 33.08	103.18 ± 22.46
*t*/*P* control group (before and after treatment)		5.709/<.001	1.610/<.001	2.651/<.001
*t*/*P* observation group (before and after treatment)		12.650/<.001	4.103/<.001	5.805/<.001
*t*/*P* inter group (after treatment)		5.663/<.001	1.486/<.001	2.255/<.001

IL-1 = interleukin-1, IL-10 = interleukin-10, TNF-α = tumor necrosis factor-alpha.

### 3.5. Adverse reactions

When comparing the overall incidence of adverse reactions between the observation group and the control group, it was found that the observation group had a significantly lower incidence rate. Specifically, the total incidence of adverse reactions in the observation group was 15.00%, compared to 22.50% in the control group. This difference was statistically significant (χ^2^ = 0.38, *P* < .01), indicating that the observation group demonstrated better outcomes in terms of adverse reactions (see Table 6).

**Table 6:** Comparison of adverse reactions between 2 groups (cases [%]).

Group	Hypotension in body position	Dizzy	Thrombocytopenia	Dry	Always occurring
Control group (n = 40)	2 (5.00)	2 (5.00)	1 (2.50)	4 (10.00)	9 (22.50)
Observation group (n = 40)	1 (2.50)	1 (2.50)	2 (5.00)	2 (5.00)	6 (15.00)*

* Comparing 2 groups, χ^2^ = 0.38, *P* < .01.

## 4. Discussion

This study demonstrated the superior efficacy of combining olanzapine and valproic acid in treating BD, particularly in improving both manic and depressive symptoms, cognitive function, and inflammatory markers, compared to olanzapine monotherapy. After 8 weeks of treatment, the observation group, which received the combination therapy, showed significantly greater reductions in BRMS and HAMD scores than the control group, with 70% of patients in the observation group achieving a therapeutic response (≥50% reduction in symptoms) for manic symptoms and 75% for depressive symptoms. In contrast, only 30% and 20% of patients in the control group showed similar reductions in manic and depressive symptoms, respectively. These results underline the enhanced therapeutic effect of the combination therapy in managing the core mood symptoms of BD, which is consistent with findings from previous studies suggesting that combining mood stabilizers and antipsychotics can be more effective than monotherapy in BD treatment.^[21–23]^

Beyond symptom improvement, the study also explored the effects of the treatment on cognitive function and inflammation, which are crucial in BD management. Cognitive impairments are a common and disabling feature of BD, affecting attention, memory, executive function, and processing speed. In our study, the observation group exhibited significant improvements in cognitive domains, such as verbal working memory and processing speed, compared to baseline, which was not seen in the control group. These findings align with existing literature, which suggests that olanzapine and valproic acid have neuroprotective effects and can help alleviate cognitive impairments in BD patients.^[24–26]^ Importantly, the combination therapy not only reduced mood symptoms but also contributed to better cognitive outcomes, highlighting its potential in addressing both emotional and cognitive components of BD.

Inflammatory markers are increasingly recognized as key contributors to the pathophysiology of BD, with evidence suggesting that neuroinflammation may exacerbate mood episodes and cognitive dysfunction. In this study, the combination of olanzapine and valproic acid was associated with a more significant reduction in IL-1 and TNF-α levels, and an increase in IL-10, compared to olanzapine monotherapy.^[27,28]^ These changes indicate a potential anti-inflammatory effect of the combination therapy, which may help to reduce neuroinflammation and improve long-term outcomes in BD. Elevated levels of pro-inflammatory cytokines, such as IL-1 and TNF-α, are implicated in neuronal damage, blood-brain barrier dysfunction, and abnormal neurotransmission, all of which contribute to the symptoms of BD. By decreasing these cytokines and increasing anti-inflammatory markers like IL-10, the combination therapy may mitigate the neuroinflammatory processes that underlie BD, providing a more holistic treatment approach.^[29,30]^

One of the most important findings of this study is the lower incidence of adverse reactions in the observation group compared to the control group. The overall incidence of adverse reactions was significantly lower in the observation group (15%) than in the control group (22.5%). This suggests that the combination therapy may be associated with better safety and tolerability, which is crucial for long-term management of BD, especially in patients who require chronic treatment. Reduced adverse effects are particularly important when considering the prolonged nature of BD treatment and the impact of side effects on treatment adherence.^[31–34]^

While these findings are promising, there are limitations that need to be addressed. First, the study’s sample size was relatively small, and future research with larger, more diverse populations is needed to confirm these results and assess the generalizability of the findings. Second, the study duration was limited to 8 weeks, which may not fully capture the long-term effects and safety profile of the combination therapy. Longer-term follow-up is necessary to assess the sustainability of the therapeutic effects and the risk of relapse or side effects over time. Finally, although we assessed key inflammatory markers, further research exploring additional biomarkers of neuroinflammation and neurodegeneration could provide deeper insights into the mechanisms underlying the observed improvements in mood and cognitive function.

## 5. Conclusions

This study demonstrates that the combination of olanzapine and sodium valproate significantly improves manic and depressive symptoms in patients with BD. Additionally, the combination therapy showed improvements in cognitive function, particularly in areas such as working memory, processing speed, and executive function. There was also a reduction in inflammatory markers, along with an increase in anti-inflammatory cytokines, suggesting a positive effect on neuroinflammation. Moreover, the combination therapy had a more favorable safety profile, with a lower incidence of adverse reactions compared to olanzapine monotherapy. These findings suggest that the combination therapy is effective in addressing both mood symptoms and cognitive impairments in BD, with potential benefits for patients experiencing significant mood fluctuations and cognitive dysfunction. Further research is needed to confirm the long-term effects and determine which patient subgroups may benefit most from this treatment approach.

## Author contributions

**Conceptualization:** Xiao Wang, Fuyu Ye, Zhen Zhang, Qigui Zhang, Guanhua Li.

**Data curation:** Xiao Wang, Fuyu Ye, Zhen Zhang, Qigui Zhang, Guanhua Li.

**Formal analysis:** Xiao Wang, Fuyu Ye, Zhen Zhang, Guanhua Li.

**Funding acquisition:** Qigui Zhang.

**Investigation:** Fuyu Ye, Zhen Zhang, Qigui Zhang.

**Methodology:** Fuyu Ye, Zhen Zhang, Qigui Zhang.

**Validation:** Xiao Wang.

**Writing – original draft:** Xiao Wang, Fuyu Ye.

**Writing – review & editing:** Xiao Wang, Fuyu Ye, Guanhua Li.
